# Double Red Cross at the Rochester Industrial Exposition

**Published:** 1911-11

**Authors:** 


### Double Red Cross at the Rochester Industrial Exposition

IN November, 1897, Captain Lomb expressed his love for children by founding the Rochester Public Health Association. For the first ten years the work done was largely along educational lines, but, after incorporating in 1908, more time was given to practical work in Clinics. Until his death in 1908, Captain Lomb bore the expense of the Association almost unaided, but since then the people of the city have risen to the demands.

The system of medical inspection in schools, the visiting nurses, the school children's dispensary, Iola Sanitorium (Monroe County Tuberculosis Hospital) and the Open Air School, all have had their origin in this Association. The Open Air School is still a part of this organization, being conducted conjointly with the School Board. The work has grown so, that now there are five active clinics: one for ear, nose and throat; one for the eye; one for nervous diseases; one for orthopedic cases; and one called the general clinic, which is the clearing house in which the children receive a complete examination and are referred to the proper clinic for treatment.

The Rochester Dental Society conducts a free dental clinic in the Association Building. The work has reached such proportions, that it has become necessary to employ a trained worker, Doctor E. G. Whipple, formerly with the State Department of Health.

The booth at the Rochester Industrial Exposition is the first public effort made by the new secretary, to show the people of the city what the association is doing, rather than to present to them any educational exhibit. A letter and a folder with half tone cuts enclosed in an envelope marked "Exposition News" is being given out to the crowds of people who visit the exposition daily. Statements carefully printed in pronounced colors giving the various cases treated in the various clinics are tastily arranged along the walls of the booth and under these charts are representations of the various clinics. This booth has been designated as the official First Aid Station for the Exposition, and the nurses of the association are in constant attendance. The necessary medical supplies are kept on hand for emergency. The double red cross, the international emblem of the fight against tuberculosis, is the distinctive feature of the exhibit.

